# Peste des Petits Ruminants Virus Fusion and Hemagglutinin Proteins Trigger Antibody-Dependent Cell-Mediated Cytotoxicity in Infected Cells

**DOI:** 10.3389/fimmu.2018.03172

**Published:** 2019-01-14

**Authors:** José M. Rojas, Daniel Rodríguez-Martín, Miguel Avia, Verónica Martín, Noemí Sevilla

**Affiliations:** Centro de Investigación en Sanidad Animal, Instituto Nacional de Investigación y Tecnología Agraria y Alimentaria (CISA-INIA), Madrid, Spain

**Keywords:** NK cells, ADCC, Morbillivirus, bluetongue virus, BTV, sheep, PPRV

## Abstract

The adaptive immune system utilizes multiple effector mechanisms to clear viral infections. Among those antibody-dependent cell-mediated cytotoxicity (ADCC) can help recognize and clear virus-infected cells. In the present work we evaluated ADCC contribution to immunity in two economically important viral diseases that affect ruminants: bluetongue and peste des petits ruminants. Immune sera obtained from sheep experimentally infected with bluetongue virus (BTV) serotype 8 or peste des petits ruminant virus (PPRV) IC'89 were used for this study. PPRV immune sera could bind to the surface of PPRV-infected ovine B cells while BTV immune sera was unable to bind to the surface of BTV-infected sheep cells but could recognize intracellular BTV antigens. BTV and PPRV immune serum ADCC potency was established using an ovine autologous cytotoxicity assay that employed an NK cell-enriched fraction as effector cells and a virus-infected B cell-enriched fraction as target cells. In this system, immune sera triggered ADCC against PPRV-infected cells, but not against BTV-infected cells. PPRV immune sera could recognize PPRV fusion and hemagglutinin proteins on the surface of transfected cells, and enhanced lysis of these cells in ADCC assays. This indicated that these viral antigens are natural ADCC targets during PPRV infection. The present work describes a novel effector immune mechanism against PPRV in the natural host that could contribute to virus clearance highlighting the importance of studying protective immune mechanisms to improve current vaccines by invoking all effector arms of immunity.

## Introduction

The adaptive immune system possesses multiple mechanisms to contain viral infections. Cellular responses are often critical for clearance of virus-infected cells. Cytotoxic T cells can recognize viral peptides presented on the cell surface and eliminate infected cells (1). The production of neutralizing antibodies is also often associated with viral disease protection (2). Binding of neutralizing antibodies on the viral particle can block virus attachment to the host cell, impair virus fusion with host cell membrane and/or eliminate virus particles though Fc dependent mechanism such as complement activation (2, 3). Non-neutralizing antibody production can also contribute to protection in some viral diseases (4). Non-neutralizing antibodies can recognize viral antigens expressed on the cell surface of infected cells and mediate cytotoxicity either through complement activation or more commonly through cell-mediated mechanisms. Antibody-dependent cell-mediated cytotoxicity (ADCC) is intrinsic to the elimination of some viral infections (5). For instance, adoptive transfer of ADCC-promoting antibodies protected mice against herpes simplex virus-2 challenge (6). ADCC activity has been linked with *in vivo* influenza A virus (IAV) protection (7) and correlated with protection in an HIV vaccine study (8). ADCC mechanism is also integral to the efficacy of monoclonal antibody infusion therapy in Ebola virus infection models (9). ADCC could therefore significantly contribute to disease clearance for some viral infections and adoptive transfer of antibodies that promote ADCC could have therapeutic potential.

ADCC is triggered when a target cell coated with antibodies is recognized by an effector cell through their Fc receptors (5). Fc receptor cross-linking on effector cells triggers a cell-mediated cytotoxicity mechanism that canonically involves effector cell cytotoxic granule release toward the infected target cell. Three types of Fc receptors are involved in ADCC mechanisms mediated by IgG binding on target cells: FcγRI (CD64) expressed on monocytes and macrophages; FcγRII (CD32) expressed on monocytes, macrophages and granulocytes; and FcγRIIIa (CD16) expressed on NK cells and on monocyte, macrophage, and γδ T cell subsets (5). In the case of viral infections, viral antigens expressed on the cell surface during infection are the most likely antibody targets for ADCC.

In the present work we wanted to assess whether ADCC mechanism could participate in the immune response and viral clearance in two economically important ruminant viral diseases of obligatory notification to the OIE: bluetongue (BT) and peste des petits ruminants (PPR). Bluetongue virus (BTV) is the causative agent of the arthropod-transmitted bluetongue disease that affects all ruminants and most severely sheep. BTV is the prototype member of the *Orbivirus* genus which belongs to the *Reoviridae* family (10). BTV genome consists of 10 segments of dsRNA that encode for 12 proteins. BTV is now endemic in Europe and present in all continents (except the Antarctica). Neutralizing antibodies are used to define BTV serotypes (11); and 27 BTV serotypes (12) [possibly 30 (13–15)] have been reported so far. BTV protection is serotype specific, and little to no protection exists across serotypes (16). As such, BTV vaccination that usually consists of inactivated virus extracts only provides serotype-specific protection.

Peste des Petits Ruminants virus (PPRV) causes PPR, a highly contagious disease that affects small ruminants and produces severe morbidity and high mortality in naïve herds, especially in goats (17). PPRV is distributed throughout Central and East Africa, the Middle East, Turkey, and India. The disease has now reached Europe doorstep with cases reported in Morocco (18), Turkey (19), and Georgia (20). PPRV is a single-stranded negative sense RNA enveloped virus from the *Morbillivirus* genus that belongs to the *Paramyxoviridae* family. The viral genome encodes for 6 structural proteins and 2 or 3 non-structural proteins (17). PPRV can produce severe immunosuppression (21) which can lead to opportunistic pathogen infections that further complicate disease recovery in affected livestock. Current PPRV vaccines consist of live attenuated strains that can still be immunosuppressive albeit to a lower extent than virulent strains.

There is therefore room to improve current vaccine strategies for both diseases. Ideally a vaccine should be safe and replicate the protective immunity that is elicited during infection. It is therefore critical to understand the exact mechanisms that drive protective immunity against these viruses on order to design more effective vaccines. Protection against both viral diseases appears to require cellular and humoral components of the adaptive immunity. Since immunity to BTV and PPRV relies partly on antibodies, some of which are likely non-neutralizing, we wanted to assess whether ADCC could contribute to disease clearance. We therefore assessed in the present work the capacity of BTV and PPRV immune sera to recognize ovine infected cells. We also measured the capacity of these immune sera to induce ADCC against infected cells and attempted to identify some of the viral antigens that are targeted by this cytotoxic mechanism. The data presented here highlight the importance for vaccines to trigger a wide range of adaptive immunity effector functions to clear viral infections.

## Materials and Methods

### Cells, Virus, and Anti-PPRV and Anti-BTV Immune Sera

293 T cells (ATCC: CRL-3216) and ovine STC cell line (an SV40-transformed sheep thymic cell line established in the laboratory) were cultured in DMEM + 10% FBS + 2 mM L-glutamine + 1% 100X non-essential amino-acids + 1 mM sodium pyruvate + 100 U/ml penicillin/100 μg/ml streptomycin. BHK-21 (ATCC: CCL-10) and Vero cells (ATCC: CCL-81) were cultured in DMEM + 5% FBS + 2 mM L-glutamine + 100 U/ml penicillin/100 μg/ml streptomycin. Vero expressing Dog-SLAM (VDS) (Dr. Parida, Pirbright, UK) were cultured in DMEM + 1% FBS + 2mM L-Gln + 1% 100X non-essential amino-acids + 1 mM sodium pyruvate + 100 U/ml penicillin/100 μg/ml streptomycin + 1μg/ml Zeocin. Ovine peripheral blood mononuclear cells (PBMC), NK cell and B cell fraction cultures were performed in RPMI + 10% FBS + 2 mM L-Gln + 1% 100X non-essential amino-acids + 1 mM sodium pyruvate + 100 U/ml penicillin/100 μg/ml streptomycin + 20 mM HEPES.

Virulent Peste des Petits Ruminants virus Ivory Coast'89 (PPRV IC'89, lineage I) isolate, vaccine PPRV strain Nigeria'75/1 (PPRV Nig'75, lineage II) (Dr. Batten, Pirbright, UK), and Bluetongue virus serotype 8 (BTV-8) Belgium'06 isolate (Prof Palmarini, University of Glasgow, UK) were used in the present work. BTV stocks were grown in BHK-21 cells and PPRV stocks were grown in VDS as described previously (22, 23). Virus stocks were tittered by plaque assays as described in Rodriguez-Calvo et al. (24). Target cells were infected at a multiplicity of infection (MOI) of three 24 h prior to cytotoxicity assays for BTV and 48 h prior to cytotoxicity assays for PPRV. These timepoints were chosen as cytopathic effects became apparent after 48 h for BTV-infected cells and 72 h for PPRV-infected.

Anti-PPRV and anti-BTV immune sera were obtained from sheep that recovered from experimental infections with virulent PPRV-IC'89 or BTV-8 strains (day 23–30 post-infection) (22, 25). Sera from the same animals prior to PPRV or BTV infection were used as naïve controls. All sheep sera were heat-inactivated prior to use (56°C, 30 min).

### Antibodies

The following antibodies were used in the present work to characterize PBMC cell populations: anti-human CD16 (clone KD1, Biorad), anti-CD3 (clone CD3-12, Biorad), anti-bovine B cell marker (clone BAQ44A, Kingfisher Biotech), anti-human CD14 (clone TÜK4, Biorad). Anti-FLAG (F7425, Sigma) and anti-HA (clone 6E2, Cell Signaling) antibodies were used to detect protein expression in transfected cells. Anti-GAPDH antibody was used as sample loading control for western blot. Anti-ovine MHC-I antibody (clone 41.17, Biorad) was used as positive control to label cells in antibody dependent cell cytotoxicity assays. Anti-PPRV-N monoclonal antibody was used 1:100 for PPRV detection (Dr. Libeau, CIRAD, Montpellier, France) in flow cytometry experiments. Anti-BTV-VP7 monoclonal antibody (VMRD; CJ-F-BTV-MAB-10 ML) was used for BTV detection. Rat anti-Mouse IgM-FITC (Clone II/41, BD biosciences), anti-mouse IgG-Alexa 488 or 647 (Thermofisher), anti-sheep-IgG Alexa 488 (Thermofisher) were used as secondary antibodies for flow cytometry, and immunofluorescence. Anti-Mouse IgG or anti-Rabbit IgG HRP-conjugated secondary antibodies (GE Healthcare) were used for western blots.

### Anti-BTV IgG ELISA

ELISA for BTV-8-specific IgG in sera were performed as described by Martin et al. (25). Briefly, Maxisorp plates (Thermofisher) were coated with serial dilutions of BTV-8 overnight at 4°C. Plates were blocked with PBS + 0.05% Tween + 2% milk for 1 h at room temperature, washed with PBS + 0.05% tween and incubated with immune or naïve serum (dilution 1:200) for 2 h at room temperature. After washing, plates were incubated for 1 h with donkey anti-sheep IgG HRP-conjugated secondary antibodies (Biorad) (dilution 1:10,000) and after extensive washing reactions were revealed with 3, 3′, 5, 5′-Tetramethyl-benzidine (TMB, Thermofisher) and stopped with sulfuric acid (3M). Absorbance was read at 450 nm on a Fluostar Omega microplate reader.

### Peripheral Blood Mononuclear Cell Isolation

Blood was obtained from healthy donor ewes housed at the Department of Animal Reproduction (INIA, Madrid Spain) and peripheral blood mononuclear cells (PBMC) purified by standard centrifugation techniques using Ficoll gradient separation as described in Rojas et al. (23).

### Cell Enrichment and NK Cell Expansion

CD14^+^ cells were depleted from PBMC using human CD14 isolation kit (Miltenyi) and according to the manufacturer's protocol. T/NK cell and B cell enrichment was obtained by nylon wool column separation (26). Briefly, CD14-depleted PBMC were incubated on medium-equilibrated nylon wool column for 45 min at 37°C, 5% CO_2_. Cells were then eluted in 2 fractions. The first 13–14 mL eluate was enriched in T and NK cells (typically >70% and 15–20%, respectively). The second eluate consisting on the cell fraction flushed out of the column was enriched in B cells (>80% typically). Separation efficiency was assessed by flow cytometry.

For NK cell expansion, CD16^+^ cells were isolated from the T/NK-enriched fraction obtained after nylon wool column separation. CD16-expressing cells were labeled with 0.5 μg/ml anti-human CD16 antibody (Clone KD1, Biorad) per 10^7^ cells for 20 min in PBS + 0.2% BSA + 2 mM EDTA at 4°C. After washing, cells were incubated with anti-mouse IgG-microbeads (Miltenyi) (20 μl/10^7^ cells) for 20 min in PBS + 0.2% BSA + 2 mM EDTA at 4°C, washed and eluted on LS column according to the manufacturer's protocol (Miltenyi). CD16^+^ isolated cells (2 × 10^5^ per well) were expanded with 2,000 IU/mL human IL-2 or 100 ng/mL ovine IL-2 for 21–28 days in 96 well U-bottom plates (27, 28). IL-2-supplemented media was replenished every 2–3 days, and cells split 1:2 every 5–7 days. CD16 expression was assessed by flow cytometry prior to use and cultures typically contained >40% CD16^+^ cells after expansion.

### Flow Cytometry and Cytotoxicity Assays

For flow cytometry staining, cells were incubated with antibody or naïve/immune serum (1:400 dilution) for 20 min on ice in PBS stain buffer (PBS + 2% FBS + 0.03% sodium azide). Cells were then washed and incubated with secondary antibodies if necessary for 20 min on ice in PBS stain buffer. Cells were fixed in 1% paraformaldehyde (PFA) prior to acquisition. For intracellular staining, cells were fixed in 4% PFA, permeabilized and stained in PBS stain buffer supplemented with 0.2% saponin. All appropriate isotype, secondary antibody alone, naïve serum, and fluorescence minus one channel controls were performed.

Flow cytometry-based cytotoxicity assays were performed as described (29, 30). Briefly target cells (B cell enriched fraction or 293T transfected cells) were labeled with PKH67 (Sigma). Target cells were incubated with naïve/immune serum (1:400 dilution) or anti-MHC-I antibody (as positive control) for 1 h, washed and co-cultured with effector cells at different ratios for 4 h. Propidium iodide (PI) was used to label dead cells and added prior to flow cytometry analysis. Spontaneous and maximum cell death (measured by addition of 0.2% saponin) was included for all target cells. Specific cell lysis was measured using the following formula: % specific lysis = (% PI^+^ target cells – % spontaneous target cell death)/(% maximum target cell death – % spontaneous target cell death) × 100. Samples were acquired on a FACScalibur flow cytometer and analysis performed with FlowJo software.

### Transfection

PPRV-P, -F, and -H genes from the PPRV Nig'75 vaccine strain were cloned into expression plasmid vectors from cDNA obtained from VDS-infected cells. PPRV-P gene was cloned in a pIRES expression plasmid expressing a FLAG epitope tag. PPRV-F and -H genes were cloned in pCAGG expression plasmids expressing a HA epitope tag. 293 T cells were transfected in 6 well plates at ~60% confluence with 1 μg plasmid DNA using Mirus T transfection reagent following the manufacturer's instructions. pCAGG-empty plasmid was used as transfection control. Transfected cells were used for protein expression or as target cells 48 h post-transfection.

### Western Blot

Cell lysates were obtained as described (31). MicroBCA protein assay kit (Thermofisher) was used to measure protein content in cell lysates and 20 μg protein per lane were resolved on 10% SDS-PAGE. After transfer to PVDF membrane, blot were probed with the appropriate primary and secondary antibodies and revealed with ECL reagent (Thermofisher). Chemiluminescence was detected using film or a Chemidoc (Biorad).

### Immunofluorescence and Confocal Microscopy

Vero cells were grown on coverslips and transfected with 1 μg plasmid DNA using Lipofectamine 3000 reagent (Invitrogen, Thermofisher) following the manufacturer's instructions. After 24 h, cells were fixed with 4% PFA for 20 min, permeabilized in PBS +0.05% Triton X-100 and blocked with Dako antibody reagent diluent. Coverslips were incubated with primary antibody diluted in Dako antibody reagent diluent overnight at 4°C. Coverslips were then incubated with secondary antibody diluted in Dako antibody reagent diluent for 45 min at room temperature. Coverslips were counterstained with DAPI prior to mounting with Prolong mounting media. Images were captured with a 63x objective using an LSM 880 confocal microscope (Zeiss). ImageJ software was used for image analysis.

### Statistical Analysis

Statistical analysis was performed with the Graphpad Prism software. Statistical tests used for data analysis are described in the figure legend. Levels of significance were as follow: ^*^p < 0.05; ^**^p < 0.01; and ^***^p < 0.001.

## Results

### IgG From PPRV but Not From BTV Immune Sera Can Recognize Viral Antigens on the Surface of Infected Cells

**Figure 1:**
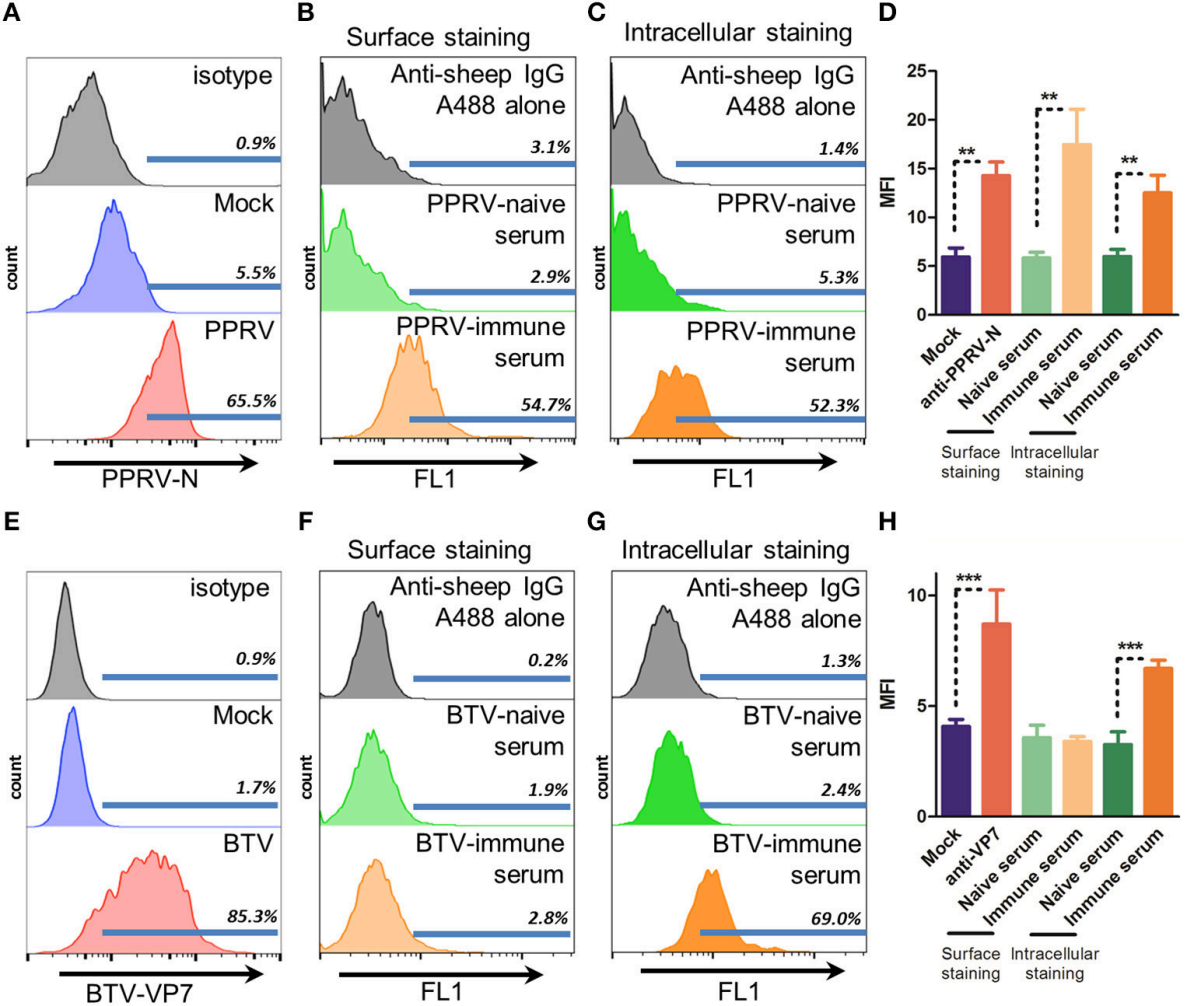
Immune sera from PPRV but not BTV bind to infected cells. **(A–D)** Representative flow cytometry histograms (*n* = *3*) of B cell enriched ovine PBMC mock-infected or infected with PPRV IC'89 at MOI 3 for 48 h and stained with **(A)** anti-PPRV-N antibody, **(B)** surface stained, or **(C)** stained intracellularly with PPRV-naïve or -immune serum or, as control, secondary antibody alone (anti-sheep IgG). **(D)** Mean fluorescence intensity (MFI) of PPRV IC'89-infected B cell enriched ovine PBMC stained with anti-PPRV-N antibody, PPRV-naïve or –immune sera. Mean ± SD MFI for 3 naive and immune sera are represented. ^**^p < 0.01 One-way ANOVA with Bonferroni's post-test. **(E–H)** Representative flow cytometry histograms of STC cells (*n* = 2–4) mock-infected or infected with BTV-8 at MOI 3 for 24 h and **(E)** stained with anti-BTV-VP7 antibody; **(F)** surface stained with BTV-naïve or -immune serum or, as control, secondary antibody alone (anti-sheep IgG); or **(G)** stained intracellularly with BTV-naïve or immune serum or, as control, secondary antibody alone (anti-sheep IgG). **(H)** Mean fluorescence intensity (MFI) of BTV-8 infected STC cells, stained with anti-BTV-VP7 antibody, BTV-naïve or -immune sera. Mean ± SD MFI for 2 to 4 naive and immune sera are represented. ^***^p < 0.001 One-way ANOVA with Bonferroni's post-test.

For ADCC to take place IgG binding to the surface of infected cells is necessary. This binding can then trigger cell death through recognition by the Fc receptors present on effector cells (5). To determine whether immune sera from PPRV IC'89 or BTV-8 infected sheep could recognize infected cells, we assessed by flow cytometry the surface binding of these sera in cells from the natural host (sheep) infected with PPRV IC'89 or BTV-8 (Figure 1). PPRV cell entry is mediated by the attachment to SLAM receptors on immune cells (32) or to nectin-4 on epithelial cells (33). We chose to assess PPRV infection in immune cells, since these are early targets during the virus infectious cycle. We enriched ovine PBMC in B cells using nylon wool fractionation and infected these cells with PPRV IC'89 at MOI 3. PPRV infection could be detected by flow cytometry in B cell-enriched ovine PBMC with anti-PPRV-N antibody (Figure 1A). PPRV immune sera could specifically bind to B cell-enriched PBMC infected with PPRV IC'89 when compared to naïve sera (Figure 1B). PPRV immune sera could also recognize intracellular PPRV antigens when staining was performed on permeabilized cells (Figure 1C). We detected an increase in infected cell mean fluorescence intensity (MFI) using three different sheep PPRV immune sera indicating that PPRV immune sera could detect surface PPRV antigens on infected cells (Figure 1D). For BTV infection, an established ovine STC cell line was used, since the virus can infect a broad spectrum of cell types *in vitro*. BTV infection (MOI 3) was detected in STC cells by flow cytometry with anti-BTV-VP7 antibody (Figure 1E). BTV-8 immune sera could not specifically bind to the surface of STC cells infected with BTV-8 (Figure 1F). BTV-8 immune sera could nonetheless specifically recognize BTV-8 infected cells when cell preparations were permeabilized (Figure 1G). The capacity of BTV-immune serum to recognize virus and infected cells was confirmed by ELISA and immunofluorescence (Supplementary Figure 1). Binding of several BTV-8 immune sera on STC infected cells was assessed to confirm this finding (Figure 1H). While immune sera could recognize intracellular BTV antigens in infected STC cells, the fluorescence intensity of these sera in surface staining was equivalent to that of naïve BTV sera. This indicated that BTV-8-infected cells only expressed on the cell surface low levels of BTV antigens recognizable by immune sera. Conversely, PPRV immune sera could detect PPRV antigens on the surface of infected cells.

### Immune Sera From PPRV Infected Sheep Induces ADCC Against Infected Cells

ADCC is classically assessed by using PBMC-derived NK cells as effector cells. ADCC is mediated by CD16 crosslinking on these cells by IgG bound to the target cell surface. CD16 is expressed on several peripheral mononuclear cell populations (NK cells, and subsets of γδ T cells, monocytes, and macrophages). NK cells however only represent a small proportion of total PBMC (typically < 5%); we therefore needed to obtain an ovine PBMC fraction enriched in fresh NK cells. Since NK cells can also mediated allogeneic killing of targets cells, we decided to assess ADCC in an autologous system to minimize non-specific killing of target cells. Thus, we also needed to obtain an autologous target cell fraction from the same donor sheep.

**Figure 2:**
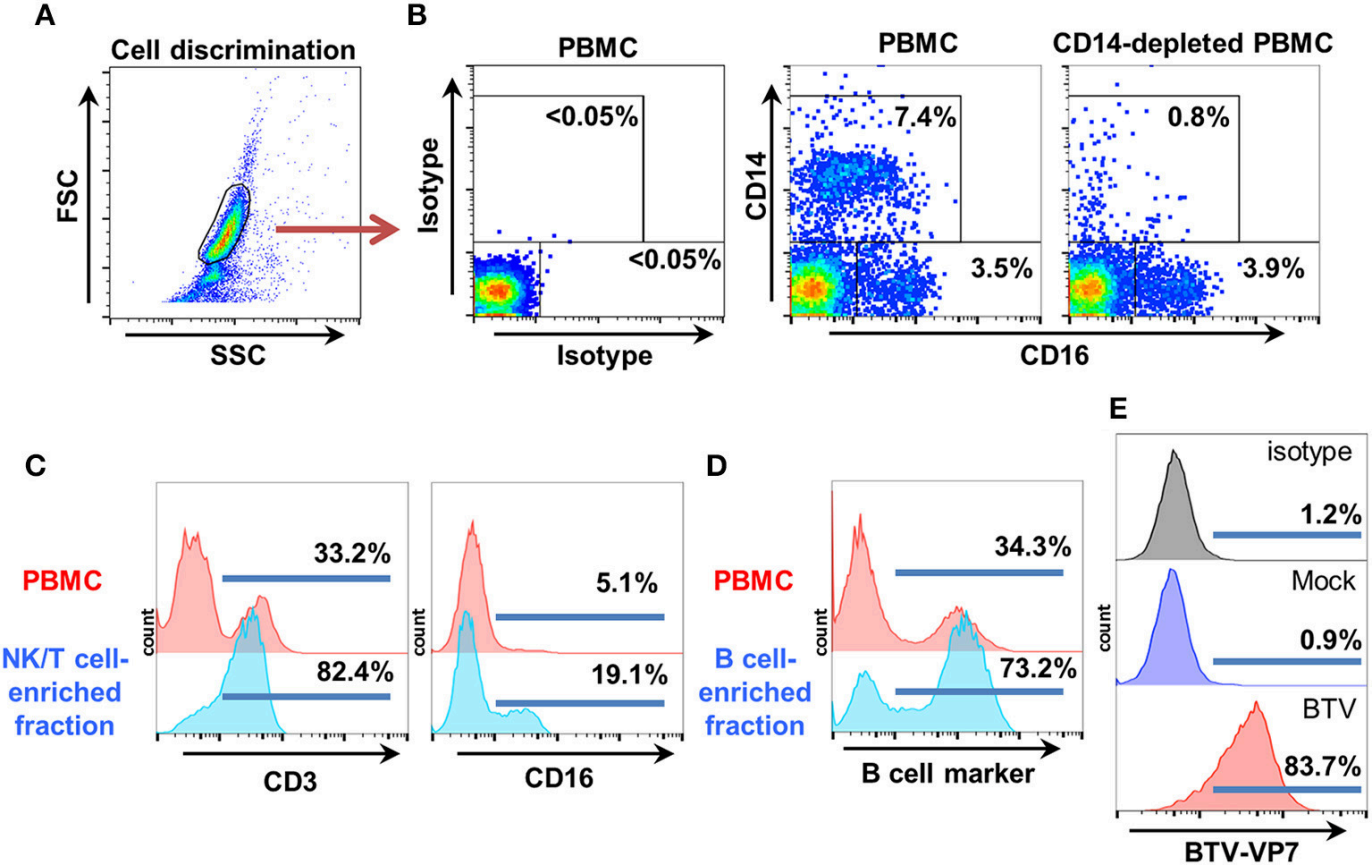
Target and effector cell isolation for ovine ADCC assays. **(A)** Ovine PBMC were obtained by standard gradient centrifugation separation. CD14^+^ cells were depleted using anti-CD14 microbeads as described by the manufacturer. Cell discrimination was established using FSC/SSC dot-plots. **(B)** Representative flow cytometry dot-plots (*n* = *4*) for CD14 and CD16 staining in PBMC and in CD14-depleted PBMC are shown. Isotype controls were used to establish CD14/CD16 gatings. **(C)** The effector fraction used in ADCC assays was obtained after elution of CD14-depleted PBMC from nylon wool columns. This fraction is enriched in NK and T cells. Representative flow cytometry histograms (*n* = *6*) after CD3 and CD16 staining in PBMC and in the NK/T cell enriched eluate are shown. **(D)** The autologous target cell fraction used in ADCC assays was obtained after flushing the cells trapped in the nylon wool column. This fraction is enriched in B cells. Representative flow cytometry histograms (*n* = *6*) for B cell marker expression in PBMC and B cell enriched eluate are shown. **(E)** The B cell-enriched fraction was mock-infected or infected with BTV-8 (MOI 3). Target cell infection with BTV-8 was monitored by flow cytometry using anti-BTV-VP7 staining.

To obtain our ovine PBMC fraction enriched in NK cells, we first removed CD14^+^ monocytes (as these cells can also express CD16) using commercial anti-CD14 antibody-coated magnetic beads and confirmed depletion by flow cytometry. FSC/SSC dot plots were used for cell discrimination and isotype control staining for gate setting (Figures 2A,B). The CD14-depleted PBMC fraction, which always contained < 1% CD14^+^ cells and retained a CD16^+^ population Figures 2B was then separated on nylon wool columns. The first fraction eluted from nylon wool column was enriched in T and NK cells. This fraction typically contained >70% CD3^+^ cells and 15–20% CD16^+^ cells, and was used as effector cells (Figure 2C). The second fraction eluted by flushing the cells trapped in the nylon wool column was enriched in B cells (>70% B cell marker^+^ cells) and used as target cells (Figure 2D). This fraction could be effectively infected with PPRV-IC'89 (Figure 1A) or BTV-8 (Figure 2E).

**Figure 3:**
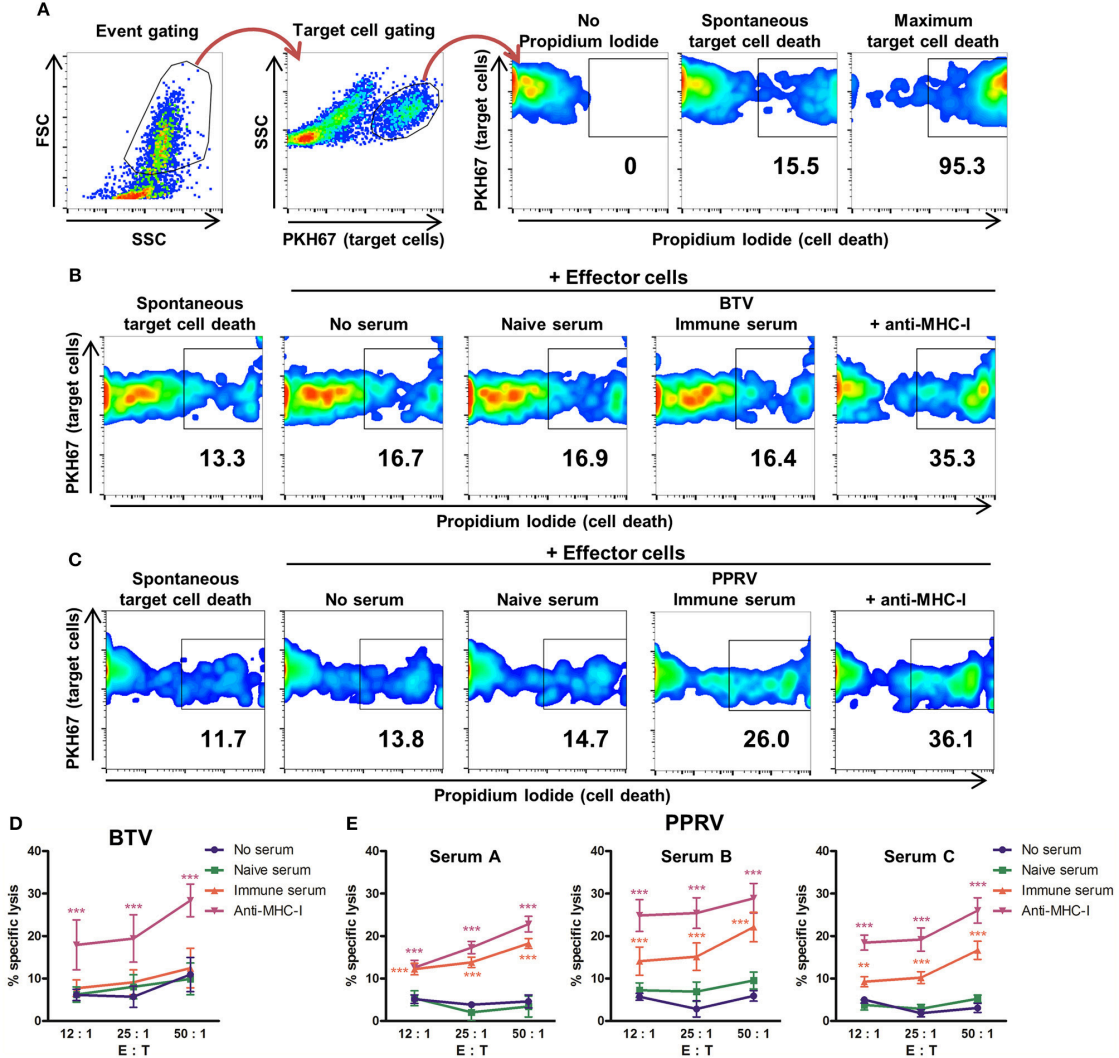
Sera from PPRV-immune sheep can mediate ADCC against PPRV infected cells. **(A)** Gating strategy for flow cytometry-based ADCC assays. FSC/SSC dot plots discriminated debris and allowed for event gating. Target cells (PKH67^+^ events) were then gated to perform the viability analysis with propidium iodide (PI) staining. Gating for dead cells was based on PI unstained samples. Maximum target cell death was measured by addition of 0.2% saponin. **(B,C)** Examples of cytometry dot-plots (duplicates from three independent experiments for each sera) used to measure specific lysis of target cells by effector cells. Representative dot plots of **(B)** BTV- or **(C)** PPRV-infected B cell enriched fraction (target cells) cultured in the absence of effector cells (spontaneous cell death) or in presence of effector cells (autologous NK/T cell fraction) after incubation with anti-MHC-I antibody as positive control, no serum, naïve serum, or PPRV-immune serum. **(D)** Mean (±SEM) specific lysis in three independent experiments of BTV-infected cells incubated with anti-MHC-I antibody as positive control, no serum, naïve, or BTV-immune serum at different effector to target cell ratio (E: T). ^***^p < 0.001 Two-way ANOVA with Dunnett's post-test (anti-MHC-I vs. controls (no serum or naïve serum). **(E)** Mean (±SEM) specific lysis in three independent experiments against PPRV-infected cells incubated with anti-MHC-I antibody as positive control, no serum, PPRV-naïve serum A, B or C, or PPRV-immune serum A, B or C at different effector to target cell ratio (E: T). ^**^p < 0.01; ^***^p < 0.001 Two-way ANOVA with Dunnett's post-test [anti-MHC-I and immune serum vs. controls (no serum and naïve serum)].

ADCC was measured by flow cytometry using target cells labeled with the membrane marker PKH67 (Figure 3A) and infected with BTV-8 (Figure 3B) or PPRV IC'89 (Figure 3C). Cell death was assessed with propidium iodide staining. The capacity of BTV immune sera to produce ADCC against BTV-infected cells was evaluated using effector and target cells obtained from three donor sheep (Figure 3D). BTV immune sera did not induce specific lysis of BTV-infected target cells when compared to cells incubated with BTV naïve sera or with no serum pre-incubation. Target cells labeled with anti-MHC-I antibodies were specifically lysed by effector cells indicating that effector cells could mediate ADCC in these experiments. The capacity of 3 PPRV immune sera (A, B, and C) to produce ADCC against PPRV-infected target cells was also evaluated with effector and target cells purified from three donor sheep PBMC (Figure 3E). All three PPRV immune sera promoted specific lysis of PPRV IC'89-infected cells when compared to target cells incubated in the absence of sera or with the matching naïve sera. PPRV immune sera therefore contain antibodies that mediate ADCC against virus-infected target cells. We were unable to detect ADCC mechanism against BTV-infected cells with immune sera.

### PPRV-F and -H Protein Expression on Infected Cells Mediates ADCC Recognition by PPRV-Immune Serum

We next set out to determine which PPRV gene products could be recognized by immune sera. PPRV particle is enveloped and contains two transmembrane proteins encoded by the viral genome: a fusion protein (F) responsible for the fusion of the viral particle with the cell plasma membrane and a hemagglutinin (H) responsible for receptor attachment on the target cell (17). PPRV infection also produces syncytia due to F protein expression on the cell surface of infected cells. Moreover, PPRV is thought to exit infected cells by budding and thus PPRV-F and PPRV-H proteins are probably present on the cell surface of infected cells (34). Antibodies against F and H proteins can be detected in individuals that recovered from Morbillivirus infection, and vaccination with recombinant adenovirus expressing these genes produces anti-PPRV antibodies and protects against virulent viral challenge (35–37). We therefore hypothesized that PPRV-F and-H could be targets for ADCC on infected cells.

**Figure 4:**
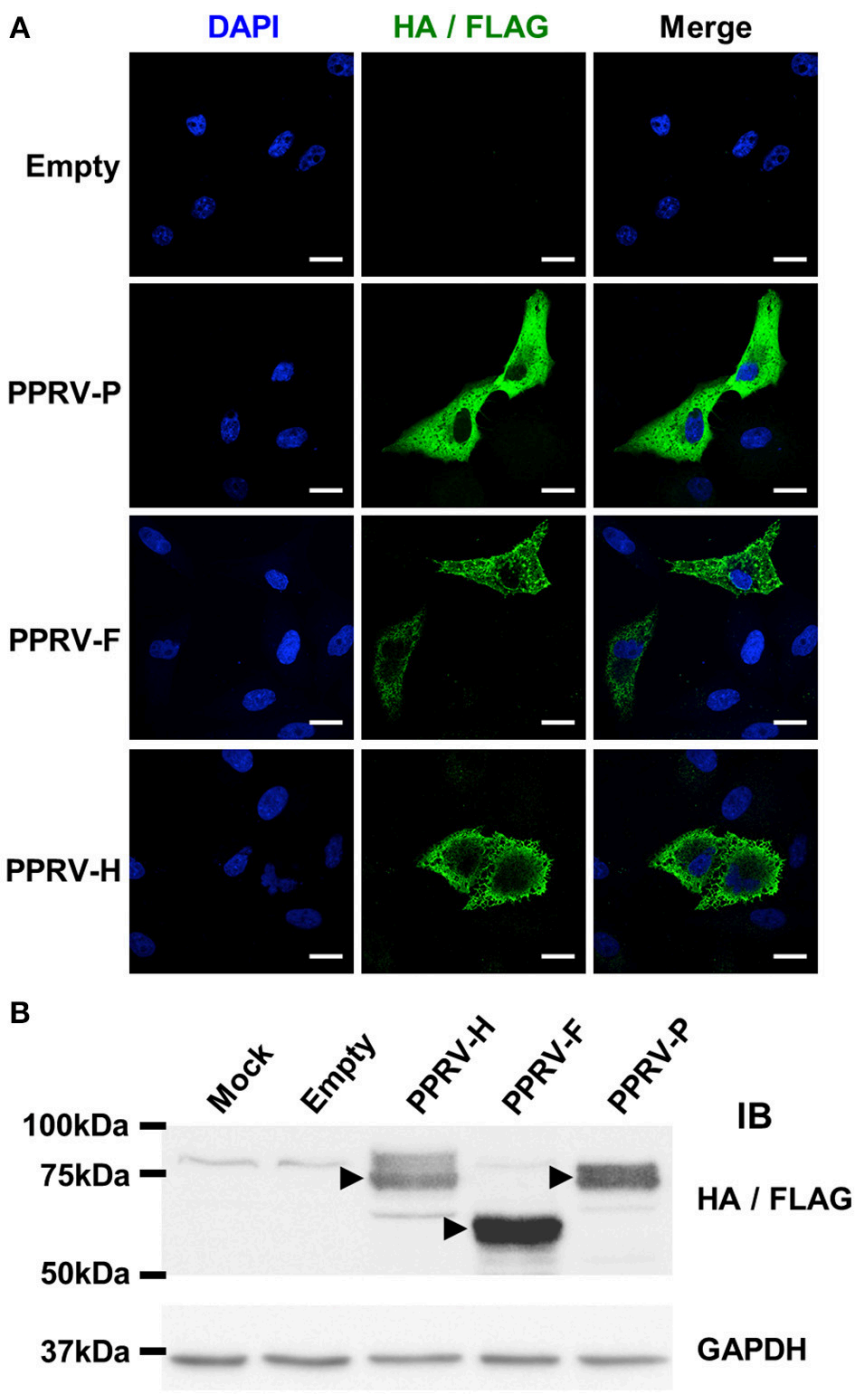
PPRV-P, -F, and -H expression in transfected cells. **(A)** Vero cells were grown on coverslips, transfected with pCAGG-Empty, -PPRV-F-HA, -PPRV-H-HA, or pIRES-PPRV-P-FLAG, and protein expression assessed using anti-tag antibodies (HA/FLAG). Nucleic acids were visualized by DAPI staining. Representative confocal images of transfected cells are shown. Scale bar = 20 μm. **(B)** 293T cells were mock-transfected (Mock) or transfected with pCAGG-Empty, -PPRV-F-HA, -PPRV-H-HA or pIRES-PPRV-P-FLAG, and cell lysate obtained. Proteins were resolved on 10% SDS-PAGE, transferred to nitrocellulose membrane and probed (IB) for protein expression using anti-tag antibodies (HA/FLAG). Membranes were also probed with anti-GAPDH antibody as loading control. Arrowheads indicate protein bands at the predicted molecular weight for PPRV-H (70 kDa), PPRV-F (59 KDa), and PPRV-P (75–79 kDa depending on phosphorylation).

To test this hypothesis, F and H cDNA obtained from PPRV-Nig'75 infected cells were cloned into pCAGG expression vectors that contain a HA epitope tag for detection. As control for a PPRV intracellular gene product, the phosphoprotein P gene cDNA from PPRV-Nig'75 was cloned in a pIRES expression vector along with a FLAG tag epitope. PPRV-F, -H, and -P protein expression was verified by immunofluorescence after transfection of Vero cells (Figure 4A). This was further confirmed by western-blot analysis of 293T cells (Figure 4B) or Vero cells (data not shown) transfected with these plasmids. HA/FLAG tag detection revealed bands at the predicted molecular weight for PPRV-H (~70 kDa), PPRV-F (~59 kDa), and PPRV-P (~75–79 kDa depending on phosphorylation).

**Figure 5:**
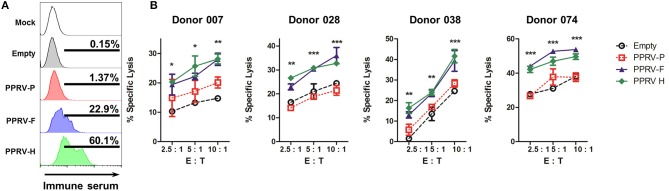
ADCC is directed to PPRV-F and -H proteins. 293T cells were mock-transfected, transfected with an empty expression plasmid or PPRV -P, -F, or -H expressing plasmids. **(A)** Transfected 293T cells were incubated with anti-PPRV immune serum and anti-sheep IgG-alexa488 secondary antibody. Sheep IgG binding to transfected cells was evaluated by flow cytometry. Representative flow cytometry histograms from three independent experiments are shown. **(B)** Transfected 293T cells were incubated with PPRV immune serum and used as target cells. ADCC was measured by flow cytometry cytotoxicity assays using IL-2-expanded ovine NK cells (>40% CD16^+^ cells) from donor sheep as effector cells. Specific lysis (mean ± SD) in ADCC assays performed in four donor sheep at different effector to target cell ratio (E: T) are shown. ^*^p < 0.05; ^**^p < 0.01; ^***^p < 0.001 Two-way ANOVA with Bonferroni's post-test (PPRV-P, -F, or -H vs. empty).

We next wanted to determine whether PPRV immune sera could detect the presence of these PPRV proteins on the surface of transfected cells. 293T transfected cells were incubated with immune sera, stained with Alexa488-conjugated secondary anti-sheep IgG antibodies, and fluorescence determined by flow cytometry (Figure 5A). PPRV immune sera could bind to PPRV-F and PPRV-H transfected cells, but not to cells mock-transfected, transfected with an empty expression vector or with a PPRV-P expression vector. PPRV immune sera can therefore recognize PPRV-F and -H proteins on the cell surface. To determine whether the specific binding of the IgG present in immune sera could trigger ADCC against PPRV-F or -H expressing cells, we used transfected cells incubated with immune sera as targets in cytotoxicity assays. IL-2-expanded ovine NK cells were used as effector cells (Figure 5B). Specific lysis was increased against PPRV-F or -H expressing target cells using NK cells isolated from four different donor sheep. Taken together, these data show that PPRV-F and -H proteins can be targets for ADCC in PPRV-infected cells.

## Discussion

ADCC responses are part of the effector immune mechanisms triggered by several viral infections (6–9, 38–40), and they are in some cases a better correlate with protection than neutralizing antibodies (8, 41). Indeed, ADCC represents one of the bases for the protection conferred by non-neutralizing antibodies. In here we evaluate the ADCC response elicited by two ruminant viruses, BTV, and PPRV. First, we show that BTV does not induce an ADCC response. Second, we describe for the first time that ovine cells infected with PPRV can be targets of an ADCC response. Moreover, our results show that PPRV proteins F and H are ADCC main targets. This data suggest that ADCC and NK cells could play important roles in modulating the course of PPRV infections.

In previous works we demonstrated that recombinant adenovirus expressing BTV or PPRV structural proteins elicited cellular and humoral immunity and protected sheep against virus challenge (25, 30, 36). In the case of BTV, the protection occurred even though only non-neutralizing anti-BTV antibodies were induced. BTV is the prototype member of the non-enveloped *Orbivirus* genus. BTV only encodes for one transmembrane protein (the viroporin NS3) which short extracellular domain is glycosylated (10). BTV particles are nonetheless tightly associated with cellular membranes as particle release has been associated with budding. It is therefore plausible that anti-BTV antibodies could detect BTV antigen on the surface of infected cells. The work presented here however indicates that BTV antigens are unlikely to be recognized by immune sera on the surface of infected cells. We show that BTV immune sera did not contain antibodies that trigger ADCC. BTV infected cells are therefore unlikely to be targeted by ADCC *in vivo*. It would be interesting in future work to assess whether antibodies targeted to the extracellular fraction of NS3 could mediate recognition of infected cells or ADCC mechanism. Given NS3 importance in BTV particle release from mammalian cells (42), induction of anti-NS3 antibodies could also form the basis for a cross-serotype reactive therapy.

PPRV is a Morbillivirus, which enveloped viral particle possesses two transmembrane structural proteins that are also expressed in infected cells (17). Here we show that cells from the natural host infected with PPRV can be targets for ADCC. Immune sera from PPRV infected sheep therefore contain anti-PPRV antibodies that trigger ADCC against infected cells. We also show that the fusion and hemagglutinin proteins encoded by PPRV can be targeted by this cytotoxic mechanism. ADCC is therefore probably involved in the clearance of PPRV-infected cells during infection. To confirm ADCC relevance in PPRV clearance *in vivo*, it would interesting in future work to isolate monoclonal antibodies that mediate this effect and determine whether their administration can provide protection against virulent challenge.

Antibodies that mediate ADCC have the potential to protect across virus serotypes. This is suggested by IAV studies where broadly cross-reactive Ab can mediate protection (43). Induction of antibodies mediating ADCC has also been correlated with better protection after IAV challenge in patients (44). Such broadly cross-reactive Abs could be expanded after seasonal IAV outbreaks and form the basis for partial protection observed. In the case of PPRV, sera obtained from PPRV IC'89 infected sheep could mediate ADCC against target cells expressing the fusion and hemagglutinin proteins from the heterologous PPRV-Nig'75 strain. ADCC-mediating antibodies against PPRV could therefore potentially contribute to protection against several PPRV lineages.

We detected ADCC against two PPRV transmembrane proteins but not to the intracellular phosphoprotein. There is limited evidence that ADCC could target intracellular viral antigens. In IAV, transfer of non-neutralizing Ab that recognize NP provide protection in murine models (45, 46). Indeed NP could transiently be expressed on the cell surface of IAV infected cells (47), and could therefore be targeted by ADCC. Evidence is nonetheless limited as to the relevance of such cytotoxic mechanism during IAV infections. It would be interesting in future work to assess whether ADCC can target PPRV nucleoprotein, as antibodies directed against this abundant PPRV antigen during infection are readily detectable in recovered animal sera.

Taken together our data indicates that BTV immune sera are unlikely to contain antibodies that mediate ADCC. Conversely, PPRV immune sera contain antibodies that trigger ADCC against infected cells. These antibodies target PPRV fusion and hemagglutinin proteins. ADCC could therefore contribute to viral clearance in PPRV infections. This works highlight the diverse immune effector mechanisms employed by the host to eliminate PPRV. Elucidating the mechanism involved in the effective clearance of the pathogen could help design more rational vaccine approaches against these economically important diseases.

## Ethics Statement

This study was carried out in in strict accordance with the recommendations in the guidelines of the Code for Methods and Welfare Considerations in Behavioral Research with Animals (Directive 86/609EC; RD1201/2005) and all efforts were made to minimize suffering. Experiments were approved by the Committee on the Ethics of Animal Experiments (CEEA) (Permit number: 10/142792.9/12) of the Spanish Instituto Nacional de Investigación y Tecnología Agraría y Alimentaria (INIA) and the Comisión de ética estatal de bienestar animal (Permit numbers: CBS2012/06 and PROEX 228/14).

## Author Contributions

JR, DR-M, MA, and VM performed the experiments. JR, DR-M, and NS analyzed the data. JR and NS designed the study and wrote the manuscript. All authors contributed to manuscript revision, read, and approved the submitted version.

### Conflict of Interest Statement

The authors declare that the research was conducted in the absence of any commercial or financial relationships that could be construed as a potential conflict of interest.
