# Supplementary figures and images for: Peste des Petits Ruminants Virus Fusion and Hemagglutinin Proteins Trigger Antibody-Dependent Cell-Mediated Cytotoxicity in Infected Cells
Source: Front Immunol. 2019 Jan 14;9:3172. doi: 10.3389/fimmu.2018.03172 (PMC6339941; doi:10.3389/fimmu.2018.03172)

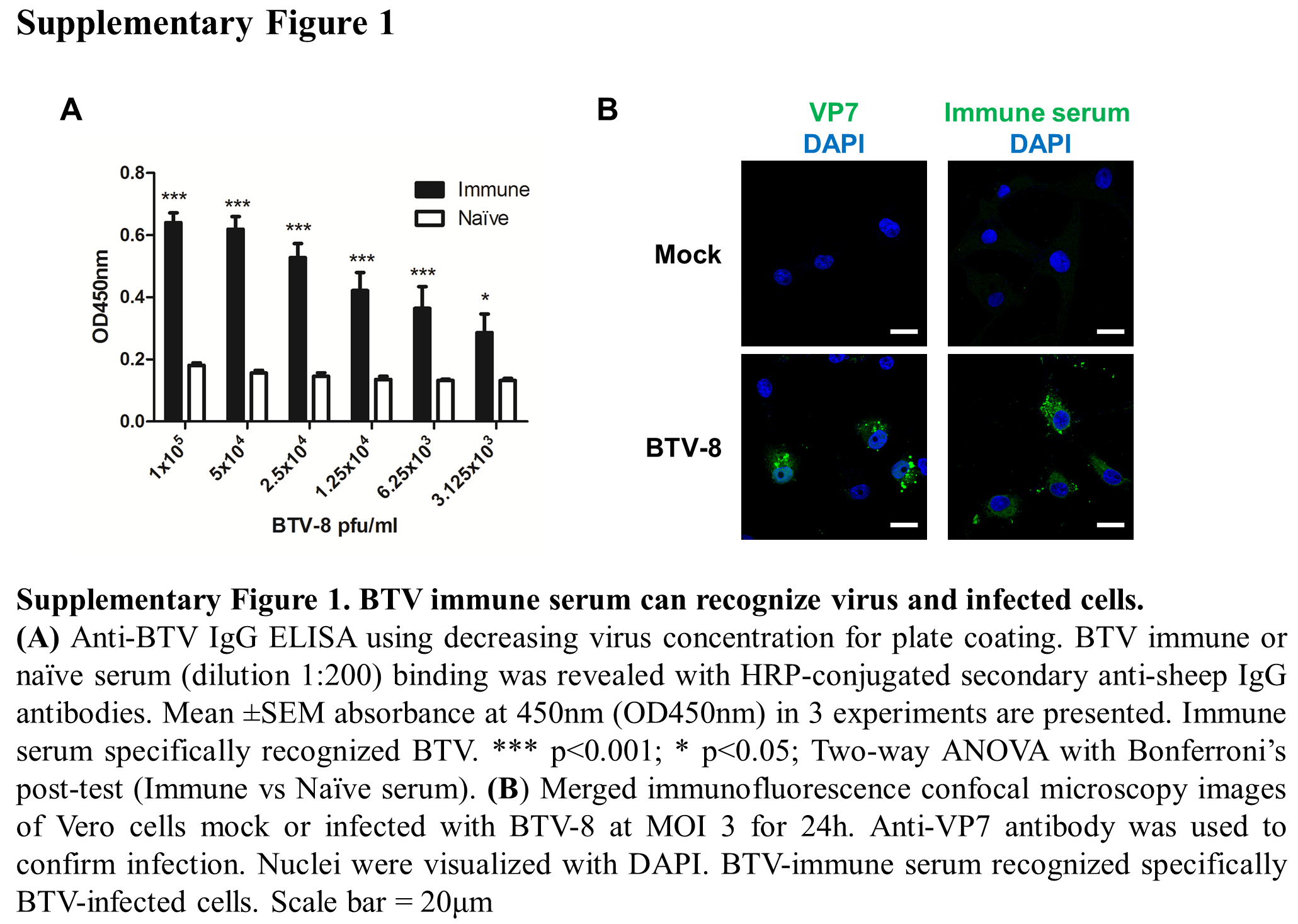

Supplement: Supplementary file 1 [file Image_1.TIF]
